# Antimicrobial activities of nano-emulsion of virgin coconut oil

**DOI:** 10.17221/57/2022-VETMED

**Published:** 2023-01-18

**Authors:** Desy Cahya Widianingrum, Himmatul Khasanah, Listya Purnamasari, Melinda Erdya Krismaputri, Seong Gu Hwang

**Affiliations:** ^1^Department of Animal Science, Faculty of Agriculture, University of Jember, Jember, Indonesia; ^2^Department of Animal Life and Environmental Science, Hankyong National University, Anseong-si, Gyeonggi-do, Republic of Korea

**Keywords:** alternative antimicrobial, nano-emulsification formula, natural resources

## Abstract

This study aimed to determine the nano-emulsion of virgin coconut oil (n-VCO) formula that can produce the best size and zone inhibition of antimicrobial activity. The VCO was formulated with the different percentages of Tween 80 (P1: 24%, P2: 25%, P3: 26%) and sorbitol (P1: 36%, P2: 35%, P3: 34%). The particle size of the n-VCO emulsion was observed under transmission electron microscopy (TEM). The antimicrobial activity test of the n-VCO was determined by a challenge test using *Salmonella* Typhi (*S.* Typhi), *Staphylococcus aureus* (*S. aureus*), and *Escherichia coli* (*E. coli*) bacteria. The data were analysed by a one-way ANOVA (*P* < 0.05). The significant data were furthermore tested by Duncan’s multiple ranges (SPSS v26.0). This study showed that the P3 formulation (26% Tween 80 and 34% sorbitol) produced the best n-VCO among all the treatments showing a particle size of 5–100 nm. Formulas P1 and P2 produced particle sizes of about 500–1 000 nm. The antimicrobial test showed that the P3 formula had a strong inhibitory effect on *S.* Typhi (7.442*** ±*** 0.52 mm), *S. aureus* (8.380*** ± ***0.49 mm), and *E. coli* (6.490 ± 0.82 mm). This study concluded that the formula of the detergent strongly influences the particle size of the n-VCO. The n-VCO has enormous potential to be used as an alternative antimicrobial.

Nanotechnologies in the livestock world have been developed for several fields, such as feed protection (Albuquerque et al. 2020), processed livestock products (Dhineshkumar et al. 2015), food packaging (Wesley et al. 2014), livestock production and health (Tatli Seven et al. 2018). A nano-emulsion is the phase of mixing the oil and water phases that blend well at a nano size of 20–200 nm. The manufacturing of nano-emulsions can be undertaken utilising oil phase disperses in the aqueous phase (oil in water emulsion o/w) or oil in water in oil (o/w/o), water in oil in water (w/o/w) and oil in water in water (o/w/w) (Marhamati et al. 2021). A nano-sized emulsion can enhance functional compounds, such as bioactive lipids, antioxidants, and antimicrobials. In the present study, we tried to apply nanotechnology to the natural ingredients of virgin coconut oil (VCO). VCO has enormous potential to be developed in the field of animal husbandry as a feed supplement (Widianingrum et al. 2019a), an antimicrobial (Widianingrum et al. 2019b), and an immunomodulator (Widianingrum and Salasia 2021). The medium-chain fatty acid (MCFA) content is known to have an essential role in the various action mechanisms of VCO as a multifunctional drug (Tangwatcharin and Khopaibool 2012; Zentek et al. 2013; Enig 2019) and can be used as an alternative to antibiotics. This study aims to determine the nano-emulsification formula of VCO (n-VCO) that can produce the best size and optimal antimicrobial activity. Our hypothesis is that the optimal formula will be found to produce the best n-VCO particles.

## MATERIAL AND METHODS

### The formula of the nano-emulsification of VCO

The materials used in this research include VCO (from the Faculty of Agricultural Technology, University of Jember, Indonesia), Tween 80 (Merck, Jakarta, Indonesia), methylparaben (PT Clariant, Ja-karta, Indonesia), sorbitol (Wintersun Chemical, Ningbo, P.R. China), propylparaben (PT Clariant, Jakarta, Indonesia), and distilled water. The nano-emulsification of VCO in this study was formulated with three treatments based on the method of Hakim et al. (2017). The composition of the formula is presented in Table 1.

**Table 1:** Nano-emulsification formula of the VCO

Ingredient	Formula (%)			
	P1	P2	P3	Control VCO
Virgin coconut oil	5	5	5	100
Tween 80	24	25	26	–
Sorbitol	36	35	34	–
Methylparaben	0.1	0.1	0.1	–
Propylparaben	0.02	0.02	0.02	–
Distilled water	100	100	100	–

Pure VCO was used as a control VCO

P1 = particle size 500–1 000 nm; P2 = particle size 500–1 000 nm; P3 = particle size 5–100 nm; VCO = virgin coconut oil

### Nano-emulsion suspension preparation

Virgin coconut oil was mixed with sorbitol (this solution is referred to as the oil phase). Methyl-paraben and propylparaben were dissolved in heated distilled water, then added with Tween 80 (this solution is referred to as the water phase). The water phase was homogenised layer using a magnetic stirrer (Thermo Fisher, Winsford, United Kingdom) at a speed of 1 100–2 000 *g*. The oil phase was gradually added to the water phase and stirred using the magnetic stirrer for 6 h at a temperature of 50 °C until a clear and transparent solution was formed (Hakim et al. 2017; with modifications, used 50 °C in the mixing process).

### Images of the nano-emulsified VCO observed by transmission electron microscopy (TEM)

Observation of the nano-emulsified VCO particles was carried out by transmission electron microscopy (TEM) at the Department of Chemistry, Faculty of Mathematics and Natural Sciences, Gadjah Mada University, Yogyakarta, Indonesia, using a JEOL JEM-1400 120 kV transmission electron microscope (JEOL, Peabody, USA).

### Susceptibility test of the nano-emulsified VCO against infectious microbes

We used microbes that cause infections and inflammatory diseases, such as *S.* *aureus*, *E.* *coli*, and *S.* Typhi to be challenged with n-VCO compared to pure VCO. Using a well, an inhibition test of nano-emulsified VCO against *S.* *aureus*, *S.* Typhi, and *E.* *coli* (strain collection: Faculty of Mathematics and Natural Sciences Collection, University of Jember, Indonesia) was undertaken with the diffusion method. A total of 50 μl of bacterial suspension was inoculated on a Mueller-Hinton agar (MHA) medium (Oxoid, Wesel, Germany). The well was made using the tip of a sterile pipette and then as much as 40 μl of nano-emulsion VCO P1, P2, P3 was placed in each well with ten repeats in each treatment. After incubation for 24 h at 37 °C, the clear zone around the well was measured using a ruler. The antimicrobial activity is based on the standards of Pan et al. (2009), namely that an inhibition zone diameter of more than 6 mm is classified as strong, 3–6 mm is classified as moderate, less than 3 mm is classified as weak, and 0 mm means there is no antimicrobial activity.

### Assessment particle size of the nano-VCO using transmission electron microscopy (TEM)

The particle size of n-VCO was descriptively compared with photographs taken by the JEOL JEM-1400 120 kV transmission electron microscope.

### Data analysis

The particle size data of the n-VCO figured by TEM was analysed descriptively. The inhibition data zones were tabulated and analysed by a one-way analysis of variance (ANOVA) and, when significant data were identified, then Duncan’s multiple range test followed. The statistical analysis was conducted using the software SPSS v26.0 (IBM, USA). The *P*-value of significant differences was set under 0.05. The inhibition zone data were also descriptively compared to the standard of Pan et al. (2009) to elucidate the power of the antimicrobial activity of the n-VCO emulsion.

## RESULTS

**Figure 1:**
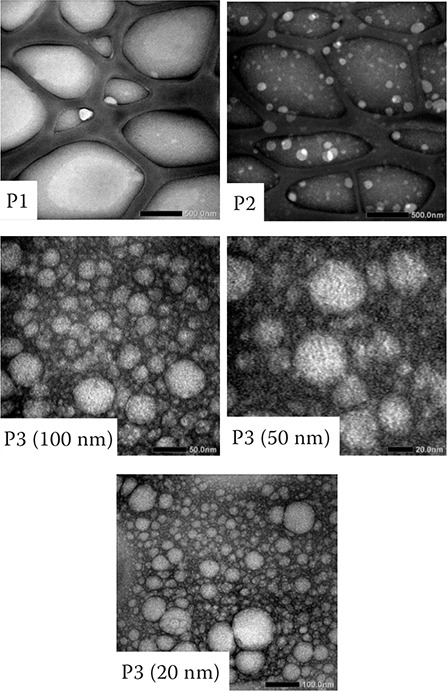
Particle size of the nano-emulsion of VCO photographed using transmission electron microscopy (TEM) P1 = particle size 500–1 000 nm; P2 = particle size 500–1 000 nm; P3 = particle size 5–100 nm; VCO = virgin coconut oil

This study showed that the P3 formulation (26% Tween 80 and 34% sorbitol) produced the best n-VCO among all the treatments showing a particle size of 5–100 nm. The formulas of P1 and P2 produced particle sizes of about 500–1 000 nm (Figure 1).

The antimicrobial test showed that the P3 formula had a strong inhibitory effect on *S.* Typhi (7.442 ± 0.52 mm), *S. aureus* (8.380 ± 0.49 mm), and *E. coli* (6.490 ± 0.82 mm). The antimicrobial activity data against those microbes are shown in Table 2.

**Table 2:** Susceptibility test of the nano-emulsion VCO against bacteria with the diffusion method

Bacteria	Treatment	Inhibition zone (mm)	Antimicrobial activity
*Salmonella* Typhi	VCO	0.000 ± 0.00^a^	no inhibition
	P1	6.630 ± 0.84^b^	strong
	P2	1.220 ± 0.599^c^	weak
	P3	7.442 ± 0.52^d^	strong
*Escherichia coli*	VCO	0.200 ± 0.47^a^	weak
	P1	5.500 ± 0.43^c^	moderate
	P2	1.420 ± 0.72^b^	weak
	P3	6.490 ± 0.82^d^	strong
*Staphylococcus aureus*	VCO	0.000 ± 0.00^a^	weak
	P1	7.270 ± 0.74^b^	strong
	P2	0.000 ± 0.00^a^	no inhibition
	P3	8.380 ± 0.49^c^	strong

Differences in the notation on the same line show significant differences (*P* < 0.05)

P1 = particle size 500–1 000 nm; P2 = particle size 500–1 000 nm; P3 = particle size 5–100 nm; VCO = virgin coconut oil

## DISCUSSION

The Tween 80 and sorbitol composition had different effects in producing the particle size in this study. The emulsion’s stability varies with the homogeniser speed, oil, and hydrophilic-lipophilic balance (HLB) values of the surfactants (Mulia et al. 2018). The mechanism for forming a nano-emulsion requires hydrophobic attraction between the surfactants and lipids so that the nano-emulsion separates the lipid-lipid. The surfactant bonds and the composition of the surfactants must be water-soluble to cover the lipid droplets’ surface (Khatri and Shao 2018). The oil phase composition is essential for the spontaneous emulsification process and determines the yield of the nano-products from both their physical and chemical properties (Bouchemal et al. 2004). The P3 treatment, having the highest Tween 80 composition, has the smallest particle size. The addition of Tween 80 in the aqueous phase composition can increase the stabilisation of the nanoparticles (Rao and McClements 2012; Koroleva et al. 2018). A previous study by Aulia (2017) that used the same formula composition in manufacturing nano avocado oil showed the best particle size with the P3 formula. In contrast, Hakim et al. (2017) reported that the nano-technique on virgin olive oil, with the same formula as our study, obtained the best treatment with the P2 composition. These findings provide information that, in addition to the composition of materials and methods, the nano-emulsion process is also influenced by the type of primary material.

Mulia et al. (2018) used VCO as the oil phase to produce a mangosteen extract nano-emulsion with the ratio of Tween 80 + Span 20 and a VCO of about 1 : 1.4 and added the oil phase into distilled water with a ratio of 1 : 1.04 (v/v). They could produce particle droplet sizes of about 181 nm. In several studies, coconut oil was used as an oil phase to produce a nano-emulsion (Suraweera et al. 2015; Samson et al. 2016; Yildirim et al. 2017). Md Saari et al. (2020) combined natural ingredients in a nano-emulsion, such as curcumin-loaded coconut oil and honey. The formula using 9% Tween 80, 1% coconut oil, 2.4% honey premix, 3% glycerol, and 0.01% polyethylene glycol as a co-solvent of curcumin produced a particle size of 15.92 nm. Although this emulsion improves the stability and skin permeability, this formula decreased the antioxidant activity of curcumin by about 7.9% compared to free curcumin (Md Saari et al. 2020).

Interestingly, the P1 and P3 nano-emulsification formulas showed strong inhibition against three bacteria, but P2 produced low inhibition against *S.* Typhi and *E.* *coli* and no inhibition to *S.* *aureus*. The difference between the P2 and P3 formulas was a 1% reduction in the sorbitol and a 1% addition of Tween 80.

The Tween 80 and sorbitol composition also showed different effects in strengthening the n-VCO antimicrobial activity. An increase in bacterial growth inhibition by nano-preparations compared to pure oil was also reported by Anwer et al. (2014). In their study, clove oil in nano-preparations gave a higher inhibitory effect than pure oil against *Bacillus subtilis*, *Staphylococcus aureus*, *Proteus vulgaris*, *Pseudomonas aeruginosa*, and *Klebsiella pneumonia*.

Pure VCO did not produce an inhibition zone in this study. Though it does not mean that the raw VCO did not have any antimicrobial activity as when VCO was challenged with *S.* *aureus* on the antimicrobial dilution method, a VCO dose of 250 μl inhibited bacterial growth (Widianingrum et al. 2019b). No inhibition zone was caused by the VCO oil in the diffusion method, so it could not directly penetrate the agar media, thus bacteria could still grow. The study of Fakhrurrazi et al. (2016) determining the potential of tamarind leaves against *Candida albicans* (*C.* *albicans*) with the diffusion method did not show an inhibition zone up to a concentration of 100%. However, when using the dilution tube method, the results showed a drastic reduction in the growth of *C. albicans* at a concentration of 10% of tamarind leaves.

In this study, the results of the pure VCO challenge test using the well diffusion method did not show an inhibition zone. However, the P1 and P3 formula nano-preparations provided a medium with strong inhibitory power against *S.* Typhi, *S.* *aureus*, and *E.* *coli* (Table 2). This illustrates the enormous potential and applicability of n-VCO as an antimicrobial that can inhibit the growth of *S.* Typhi, *S.* *aureus*, and *E.* *coli* bacteria rather than using pure VCO. Yildirim et al. (2017) compared the nano-emulsions prepared by the low-energy method (as used in our study) and the high-energy method (ultra-turrax, micro-fluidisation, ultrasonication) resulting in inhibition zones that were not significantly different to all the used methods. All the methods had strong inhibition against *E. coli*. Besides the utilisation, the other advantage of using nano-emulsion preparations is that they are easy to apply, which means the product can be straightforwardly used as external medicine in dealing with livestock health problems or for other treatments.

VCO contains many MCFAs, including lauric acid (LA) and its monoglyceride form. Monolaurin (monoglyceride form of LA) makes VCO effective in its mode of action against pathogenic microorganisms (Tangwacharin and Khopaibool 2012; Dayrit 2015; Abbas et al. 2017). It has been reported that VCO contains more than 70% medium-chain triglycerides (MCTs) (Isyanti and Sirait 2021). The term MCT refers to triglycerides, composed of a glycerol backbone and three saturated fatty acids with a chain length of 8–12 carbons. MCTs are low in molecular weight and highly soluble in biological fluids. These properties make MCTs unique because they are not metabolised through the intestinal walls like other fats, but are metabolised in the liver. MCTs are not deposited as fat, but they are burned into energy instead, so they are a substance that has a beneficial effect on the health or the body’s metabolism process (Carandang 2008; Dayrit 2014; Nasir et al. 2018).

In conclusion, the concentration of sorbitol and Tween 80 affects the particle size and antimicrobial activity of VCO-based nano-emulsions. The P3 formulation produced the smallest particle size and most potent antimicrobial activity against several pathogenic bacteria.

Further research on the biophysical properties of n-VCO needs to be elaborate upon to enhance the understanding of the n-VCO formula’s effect, including the correlation of Tween 80 and the antimicrobial activity and stability. The application of n-VCO in practical studies, such as natural antimicrobials in livestock, food technology, and pharmaceuticals, can be considered.
